# The association between multi-inflammatory index and long-term mortality in post-myocardial infarction patients treated with percutaneous coronary intervention

**DOI:** 10.3389/fcvm.2025.1590658

**Published:** 2025-06-25

**Authors:** Sangho Hyun, Jaeho Seung, Kwan Yong Lee, Sang Hyun Kim, Myunhee Lee, Andrew H. Yoon, Wonjae Lee, Byung-Hee Hwang, Eun-Ho Choo, Chan Jun Kim, Jin-Jin Kim, Ha-Wook Park, Gyu Chul Oh, Yun Seok Choi, Youngkeun Ahn, Kiyuk Chang

**Affiliations:** ^1^ College of Medicine, The Catholic University of Korea, Seoul, Republic of Korea; ^2^ Cardiovascular Center and Cardiology Division, Seoul St. Mary’s Hospital, The Catholic University of Korea, Seoul, Republic of Korea; ^3^ Cardiovascular Research Institute for Intractable Disease, College of Medicine, The Catholic University of Korea, Seoul, Republic of Korea; ^4^ School of Public Health, San Diego State University, San Diego, CA, United States; ^5^ Carle Illinois College of Medicine, University of Illinois at Urbana-Campaign, Urbana, IL, United States; ^6^ Department of Internal Medicine, Seoul St. Mary’s Hospital, The Catholic University of Korea, Seoul, Republic of Korea; ^7^ Cardiovascular Center and Cardiology Division, Uijeongbu St. Mary’s Hospital, The Catholic University of Korea, Uijeongbu, Republic of Korea; ^8^ Department of Cardiology, Bucheon Sejong Hospital, Bucheon, Republic of Korea; ^9^ Department of Cardiology, Cardiovascular Center, Chonnam National University Hospital, Gwangju, Republic of Korea

**Keywords:** inflammatory marker, myocardial infarction, biomarker, prognosis, C-reactive protein

## Abstract

**Background:**

Inflammation plays a crucial role in the pathophysiology of acute myocardial infarction (AMI), and various inflammatory markers have been associated with patient outcomes. The multi-inflammatory index (MII) has emerged as a potential prognostic indicator, but its relationship with AMI mortality remains unclear.

**Methods:**

We analyzed 8,414 patients with successfully revascularized AMI. The subjects were divided into a high MII group (*n* = 3,708) or a low MII group (*n* = 4,706) using the MII score at admission. The MII score was calculated using the initial serum neutrophil, lymphocyte, and C-reactive protein (CRP). The primary and secondary outcomes were all-cause mortality and major adverse cardiac and cerebrovascular events (MACCE).

**Results:**

Over a median follow-up of 5.13 years, the high MII group showed significantly higher incidences of all-cause mortality and MACCE than the low MII group (*p* < 0.001, each). Multivariate Cox regression identified a high MII score as an independent predictor of all-cause mortality and MACCE [adjusted hazard ratio (HR) 1.71; 95% confidence interval (CI) 1.55–1.89; *p* < 0.001, HR 1.53; 95% CI 1.40–1.67; *p* < 0.001]. MII score had statistically higher discriminative ability for predicting all-cause mortality than the conventional inflammatory marker, CRP (C-index 0.662; 95% CI 0.648–0.677 vs. 0.646; 95% CI 0.632–0.661, *p* < 0.001). The predictive accuracies of traditional clinical factor discrimination and reclassification for mortality were significantly improved upon the addition of high MII score (C-index 0.791 vs. 0.780; 95% CI 0.780–0.803; *p* < 0.001, NRI 0.018; 95% CI 0.014–0.021; *p* < 0.001).

**Conclusion:**

In the AMI cohort, a high MII score was strongly associated with long-term mortality and MACCE.

## Introduction

Cardiovascular disease is one of the leading causes of death, accounting for around 30% of all fatalities (1). Atherosclerosis, previously regarded as merely a cholesterol accumulation disorder, is now understood to be a continuous, inflammatory, and dynamic process which involves multiple inflammatory cells and signaling molecules (2, 3). To identify these inflammatory molecules or responsible immune cells and, ultimately, to find novel therapeutic targets, a number of clinical studies have been conducted on the myocardial infarction (MI) population. Ridker et al. demonstrated in Canakinumab Anti-inflammatory Thrombosis Outcome Study (CANTOS) that canakinumab, a monoclonal antibody that targets interleukin-1β, significantly lowers the recurrence rate of cardiovascular events in patients with MI (4). The CANTOS trial included patients with a history of myocardial infarction and elevated baseline C-reactive protein (CRP). The study raised the possibility that modulating certain stages of inflammation may influence the development of cardiovascular disease. However, CRP is a non-specific marker that can be elevated in a variety of situations, leaving a clinically unmet need to find a better marker.

Recent research has shown that patients with pronounced early-phase inflammation after acute myocardial infarction (AMI) have a worse prognosis (5). This suggests that a superior prognostic marker may exist within commonly measured inflammatory indicators, such as inflammatory cell counts. This is plausible given prior studies linking these readily available biomarkers and indices to increased coronary artery disease (CAD) risk (6–8). For example, the Neutrophil-to-Lymphocyte Ratio (NLR) and Platelet-to-Lymphocyte Ratio (PLR) have been evaluated to be potential predictors for worse clinical outcomes in post-MI patients. Additionally, recent research suggests using the C-reactive protein-to-albumin ratio (CAR) or a combination of CRP with NLR to form a reliable multi-inflammatory index (MII) (9–11). However, the prognostic efficacy of MII in relation to cardiovascular disease or events has yet to be examined. We therefore aimed to evaluate the predictive capabilities of MII for long-term clinical outcomes among patients who have undergone percutaneous coronary intervention (PCI) for MI.

## Materials and methods

### Study protocols, population selection, and grouping

**Figure 1:**
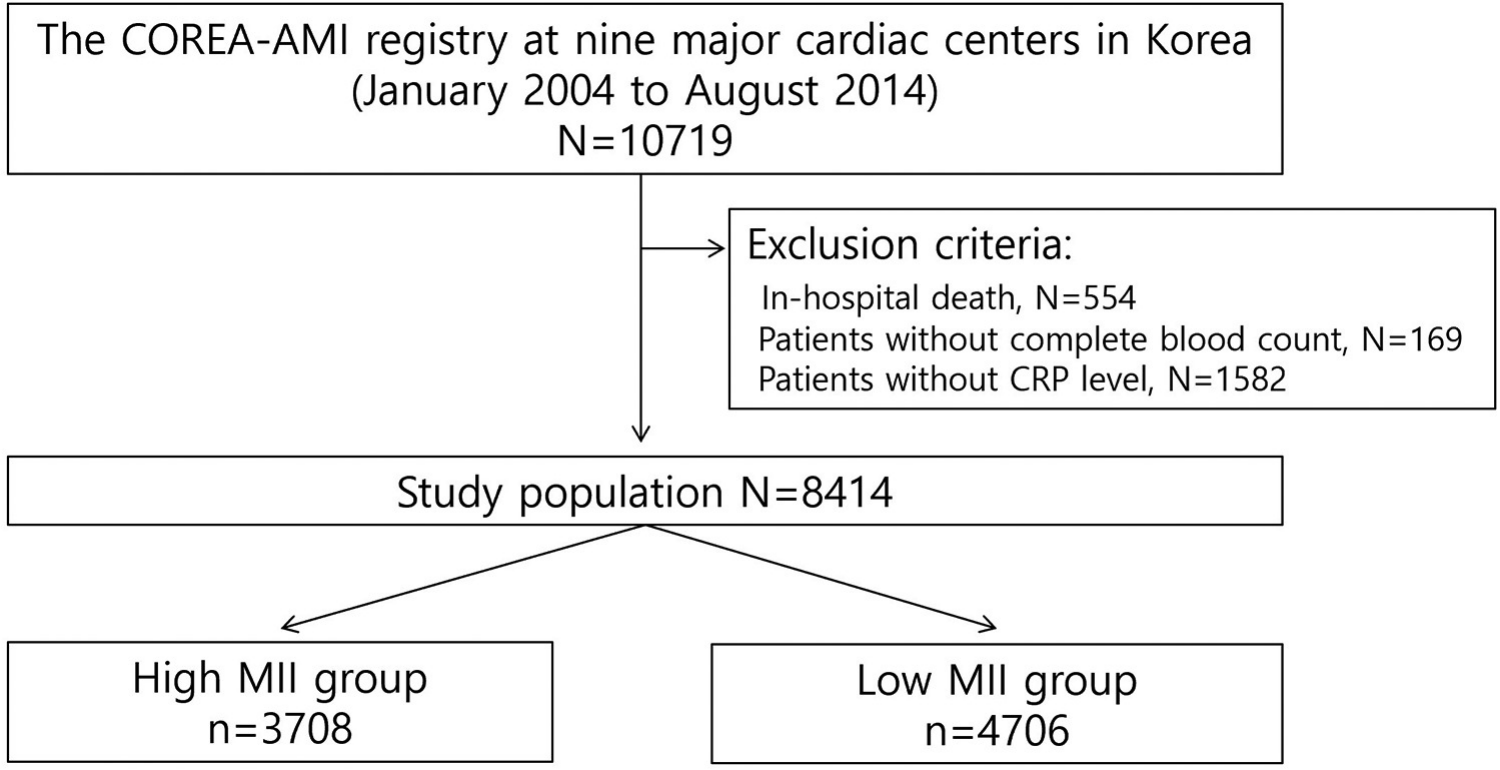
Study flow-chart.

We analyzed data from the COREA-AMI (Cardiovascular Risk and Identification of Potential High-Risk Population in Acute Myocardial Infarction) registry, which includes the long-term clinical outcomes of consecutive patients with acute MI across nine high-volume PCI centers in Korea from January 2004 to August 2014. The COREA-AMI study was conducted in accordance with the Declaration of Helsinki. This observational study was approved by the institutional review board of our institution (IRB No.XC15RSMI0089 K), and was performed in accordance with the Strengthening the Reporting of Observational Studies in Epidemiology guidelines. Written informed consent was waived by the Catholic Medical Center Central Institutional Review Board. The COREA-AMI registry is registered on ClinicalTrials.gov (NCT02806102). All 10,719 patients included were over 20 years of age and had undergone PCI for acute MI. Patients who experienced in-hospital death (*n* = 554), or did not have complete blood count (CBC) (*n* = 169) or CRP (*n* = 1,582) data were excluded. Measurement of CRP was conducted using a latex-enhanced high-sensitivity CRP immunoassay (Cobas Integra; Roche Diagnostics) across all participating centers. Consequently, a total of 8,414 patients were selected for the analysis (Figure 1).

Patients were classified into high MII and lowMII groups based on MII scores, which were derived from the initial serum levels of neutrophil count, lymphocyte count, and CRP, all measured at the time of admission prior to PCI. The optimal cutoff value of MII in our study was 16.80, which was determined using Receiver Operating Characteristic (ROC) curve analysis with the Youden index. An MII level of over 16.80 was defined as high MII in our study.

### Treatment and data collection

Patients in this study were managed in accordance with current standard acute MI guidelines. Long-term treatment of aspirin or a P2Y_12_ inhibitors were prescribed following PCI, and loading doses for each agent were administered before or during procedure, if indicated. The revascularization techniques, selection of devices, and adjunctive antithrombotic therapy were at the discretion of individual operators. Furthermore, patients were initiated on guideline-directed medical therapy (GDMT) after PCI, which included antiplatelet agents, statins, beta-blockers, and renin-angiotensin-aldosterone system inhibitors as per the prevailing practice guidelines. This analysis did not include the use of mineralocorticoid antagonists due to a lack of data and a low percentage of usage.

Trained reviewers, who were unaware of the results, collected pertinent patient information by conducting reviews of hospital charts and conducting phone interviews. After ensuring the removal of personally identifiable information, the gathered data was organized within a web-based system. An independent clinical event-adjudicating committee reviewed all reported outcome data from participating centers. Mortality was confirmed through the process of verifying disqualification from the National Health Insurance Service, Korea's universal healthcare program. The final dataset was managed by independent statisticians at the clinical research coordinating center and sealed securely by the clinical research associate with a code.

### Study endpoints and definitions

The primary endpoint of this study was all-cause death. The secondary endpoints included the occurrence of major adverse cardiac and cerebrovascular events (MACCE), which was defined as a composite of all-cause death, MI, or cerebrovascular accident (CVA). The definition of MI was based on the Third Universal Definition of Myocardial Infarction. CVA was defined as a focal loss of neurological function caused by either an ischemic or hemorrhagic event, resulting in residual symptoms lasting at least 24 h or leading to death. The time to event was measured starting from the initial MI. Revascularization was excluded from the composite MACCE definition to focus on hard clinical endpoints such as mortality, MI, and stroke that are less subject to treatment bias or physician discretion. Unlike death, MI, or stroke which are objective and clearly adverse, revascularization may reflect variations in clinical practice, institutional protocols, or patient preference rather than true worsening of disease. Additionally, in observational studies, revascularization may be confounded by access to care or operator judgment, potentially diluting the predictive value of the composite endpoint.

The calculation of inflammation indices was performed in the following manner: NLR = Neutrophil count/lymphocyte count, PLR = Platelet count/lymphocyte count, Systemic inflammatory index (SII) = NLR × platelet count, Multi-inflammatory index (MII) = NLR × CRP.

### Statistical analysis

Continuous variables were summarized as mean ± SD and compared using an unpaired t-test. Categorical variables were presented as a number (percentage) and analyzed using the *χ*^2^ test or Fisher's exact test. Missing data were addressed using a complete case analysis approach. Patients with missing values for any variable included in the relevant analyses were excluded from those specific analyses. The proportion of missing data was low for most variables (<0.1%), and the authors assumed the data were missing at random.

To determine the optimal cut-off values for each inflammatory index in predicting all-cause mortality, we performed ROC curve analysis. The Youden index (*J*), defined as *J* = sensitivity + specificity − 1, was calculated for each point on the ROC curve, and the threshold corresponding to the maximum Youden index was selected as the optimal cut-off. This approach identifies the point that maximizes the overall diagnostic effectiveness by balancing sensitivity and specificity. The resulting cut-off values were as follows: PLR (120.11), NLR (3.58), CRP (5.70 mg/L), SII (767.98), and MII (16.80). The area under the ROC curve was calculated to quantify the discriminatory performance of the inflammatory indices. DeLong's test was used to compare the predictive abilities of the inflammatory indices by comparing their area under curve (AUC) values.

Kaplan–Meier analyses and the log-rank test were used to visualize and compare cumulative incidence curves. Cox proportional hazard analyses were performed to identify independent risk factors, calculating hazard ratios with 95% confidence intervals. In the multivariable Cox proportional hazards model, variables were adjusted if they exhibited significant differences between the two groups in the univariable analysis for baseline characteristics. The adjusted variables included old age, gender, high blood pressure, diabetes mellitus, smoking, previous coronary artery bypass graft, previous stroke, anemia, chronic kidney disease, chronic lung disease, as well as medications prescribed at discharge. Also, as a sensitivity analysis, we constructed propensity score (PS)-matched cohorts comparing patients with low vs. high MII. Propensity scores were calculated using logistic regression taking into account the variables that significantly differed between the groups, including age, sex, hypertension, diabetes, smoking status, severe anemia, renal disease, chronic lung disease, history of coronary artery bypass grafting (CABG), stroke, and discharge medications. A 1:1 greedy nearest-neighbor matching algorithm without replacement and with a caliper width of 0.2 standard deviations was applied, yielding 3,433 matched pairs from the high and low MII groups. Cox proportional hazards regression was performed on the matched cohort. Additionally, an inverse probability weighting (IPW) approach was employed, incorporating the inverse of the propensity score into the Cox model.

ROC analysis was performed to evaluate the ability of MII to predict mortality in patients with AMI. The overall predictive performance of MII was compared to that of CRP, a conventional inflammatory marker. To evaluate the incremental predictive value of high MII for all-cause mortality and MACCE, four prediction models were constructed (two for each outcome). Model A (for mortality) and Model A' (for MACCE) included traditional risk factors that differed significantly between the high and low MII groups. Model B (for mortality) and Model B' (for MACCE) extended their respective Model A/A' by incorporating high MII levels. Included traditional risk factors were old age, gender, HBP, DM, anemia, CKD, smoking history, history of CABG, stroke, and chronic lung disease. Subgroup analyses were carried out based on age, sex, hypertension, diabetes, and smoking using the Cox regression analysis. The analysis for each measure was conducted using R version 4.1.2 (R Foundation for Statistical Computing, Vienna, Austria). Statistical significance was determined by a *p*-value of less than 0.05.

## Results

A total of 8,414 acute MI patients who underwent successful PCI and had CBC and CRP findings were analyzed (Figure 1). Table 1 provides a summary of the baseline characteristics of the study participants. ROC curve analysis using the Youden index determined the optimal cut-off values for the inflammatory indices. These cut-off values were: PLR (120.11), NLR (3.58), CRP (5.70), SII (767.98), and MII (16.80). Comparison of the AUC values revealed that MII had a statistically significantly larger C-index than all other indices, including CRP, which had the second largest C-index (Table 2). Based on the MII cut-off value of 16.80, patients were divided into two groups: a high MII group (*n* = 3,708) and a low MII group (*n* = 4,706). At this cut-off, MII demonstrated a sensitivity of 68.9% and a specificity of 55.6%.

**Table 1:** Baseline characteristics of study population.

Variables	Total (*n* = 8,414)	High MII group (*n* = 3,708)	Low MII group (*n* = 4,706)	*p*-value
Age (yrs)	63.3 ± 12.7	65.5 ± 12.7	61.5 ± 12.5	<0.001
Gender, female (%)	2,334 (27.7)	1,192 (32.1)	1,142 (24.3)	<0.001
HBP (%)	4,389 (52.2)	2,082 (56.1)	2,307 (49.0)	<0.001
DM (%)	2,658 (31.6)	1,363 (36.8)	1,295 (27.5)	<0.001
Dyslipidemia (%)	1,387 (16.5)	598 (16.1)	789 (16.8)	0.433
Smoking (%)	4,639 (55.1)	1,899 (51.2)	2,740 (58.2)	<0.001
Previous MI (%)	338 (4.0)	155 (4.2)	183 (3.9)	0.499
Previous PCI (%)	610 (7.2)	283 (7.6)	327 (6.9)	0.230
Previous CABG (%)	41 (0.5)	26 (0.7)	15 (0.3)	0.012
Previous Stroke (%)	582 (6.9)	335 (9.0)	247 (5.2)	<0.001
Cancer (%)	279 (3.3)	127 (3.4)	152 (3.2)	0.620
Chronic lung disease (%)	208 (2.5)	113 (3.0)	95 (2.0)	0.003
Chronic liver disease (%)	81 (1.0)	27 (0.7)	54 (1.1)	0.051
Anemia (%)	2,983 (35.5)	1,797 (48.5)	1,186 (25.2)	<0.001
CKD (%)	2,026 (24.1)	1,214 (32.7)	812 (17.3)	<0.001
Laboratory findings				
White blood cell (10^9^/L)	11.4 ± 6.3	12.6 ± 7.2	10.4 ± 5.4	<0.001
Neutrophil (10^9^/L)	7.3 ± 9.3	8.7 ± 13.5	6.1 ± 3.2	<0.001
Lymphocyte (10^9^/L)	2.8 ± 2.0	2.5 ± 2.0	3.0 ± 2.0	<0.001
Platelet (10^9^/L)	232.8 ± 66.4	234.1 ± 71.7	231.8 ± 62.0	0.120
C-reactive protein (mg/L)	35.8 ± 132.8	78.2 ± 191.8	2.3 ± 3.0	<0.001
NLR	3.8 ± 4.6	5.2 ± 6.1	2.7 ± 2.4	<0.001
PLR	118.6 ± 95.0	141.1 ± 115.8	100.8 ± 69.6	<0.001
SII	894.1 ± 1,188.3	1,221.3 ± 1,589.1	636.2 ± 619.8	<0.001
MII	168.4 ± 1,121.8	376.0 ± 1,667.1	4.8 ± 4.3	<0.001
Medication at discharge				
Aspirin (%)	8,281 (98.4)	3,638 (98.1)	4,643 (98.7)	0.045
Clopidogrel (%)	7,295 (86.7)	3,360 (90.6)	3,935 (83.6)	<0.001
Potent P2Y12 inhibitor (%)	1,073 (12.8)	312 (8.4)	761 (16.2)	<0.001
Oral anticoagulant (%)	222 (2.6)	124 (3.3)	98 (2.1)	<0.001
Beta-blockers (%)	7,394 (87.9)	3,216 (86.7)	4,178 (88.8)	0.004
Statins (%)	8,158 (97.0)	3,576 (96.4)	4,582 (97.4)	0.014

Data are presented as mean ± standard deviation for continuous variables and *n* (%) for categorical variables. Anemia defined as hemoglobin <13.5 mg/dl for males and <12 mg/dl for females. CKD defined as estimated glomerular filtration rate <60 ml/min/1.73 m^2^. HBP indicates high blood pressure; DM, diabetes mellitus; MI, myocardial infarction; PCI, percutaneous coronary intervention; CABG, coronary artery bypass graft; CKD, chronic kidney disease; NLR, neutrophil to lymphocyte ratio; PLR, platelet to lymphocyte ratio; SII, systemic immune-inflammatory index; MII, multi-inflammatory index.

**Table 2:** Discrimination ability of inflammatory indices for predicting all-cause mortality.

Models		C-index	95% CI	*p*-value	Model comparison
SII	NLR × platelet count	0.581	0.566–0.596		
PLR	Platelet count/lymphocyte count	0.586	0.570–0.602	0.307	(vs. SII)
NLR	Neutrophil count/lymphocyte count	0.595	0.579–0.610	0.152	(vs. PLR)
CRP	High sensitivity C-reactive protein	0.646	0.632–0.661	<0.001	(vs. NLR)
MII	NLR × CRP	0.662	0.648–0.677	<0.001	(vs. CRP)

SII, systemic inflammatory index; PLR, platelet-lymphocyte ratio; NLR, neutrophil-lymphocyte ratio; CRP C-reactive protein; MII multi-inflammatory index.

The high MII group patients were characterized by older age, a lower proportion of smokers, and higher percentages of females, high blood pressure, diabetes mellitus, chronic lung disease, anemia, chronic kidney disease, and a history of CABG and stroke compared to the low MII group. In addition, high MII group patients demonstrated elevated levels of white blood cell (WBC), neutrophil, and CRP while they exhibited lower lymphocyte levels. Regarding medication, a smaller proportion of these patients took aspirin, potent P2Y_12_ inhibitors such as ticagrelor and prasugrel, beta-blockers, and statins while a higher percentage of them took clopidogrel and oral anticoagulant prescriptions at the time of discharge.

**Figure 2:**
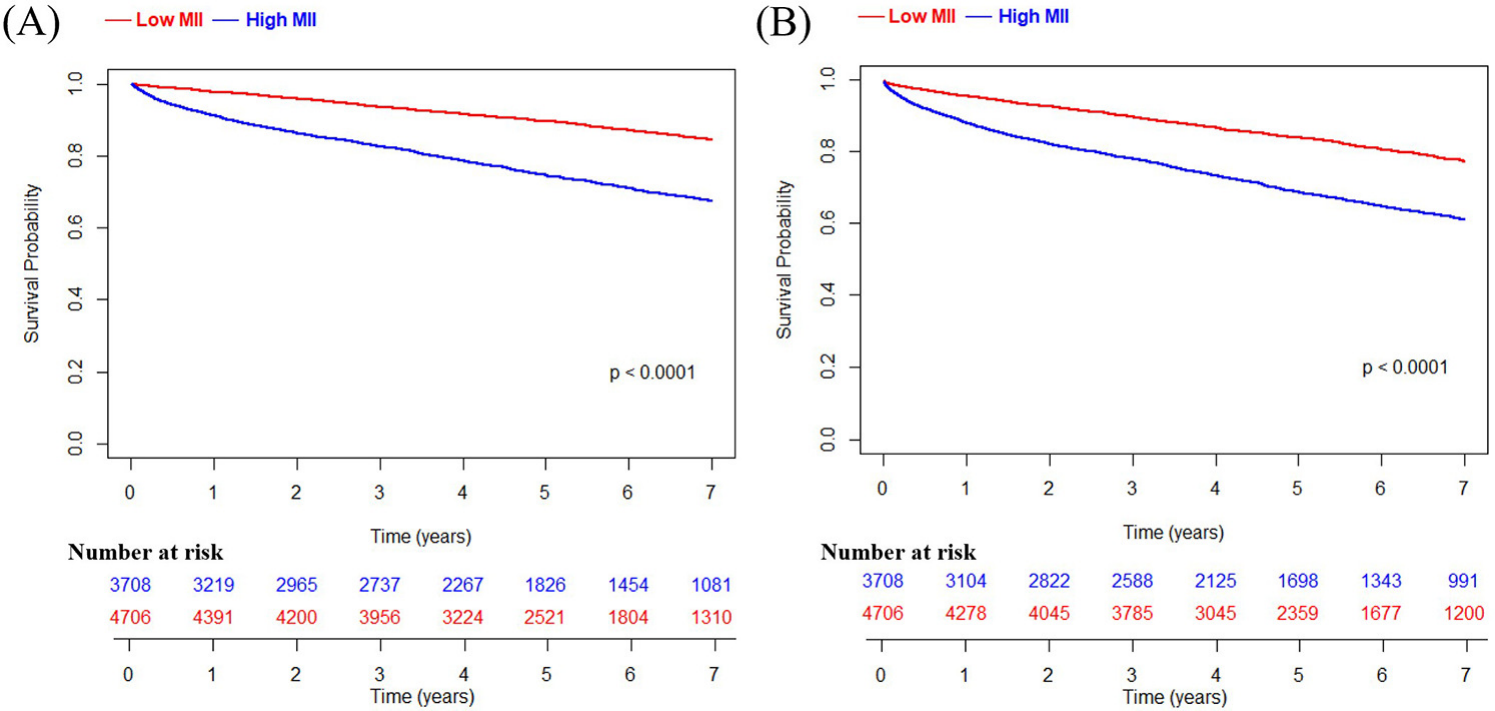
Kaplan meier survival curve of the high MII group and the low MII group. **(A)** overall death; **(B)** MACCE, which includes overall death, MI, and stroke. MII indicates multi-inflammatory index; MACCE, major adverse cardiac and cerebrovascular events; MI, myocardial infarction.

During the follow-up period, patients with high MII group experienced significantly higher rates of mortality, MACCE, and MI (Table 3). The Kaplan–Meier survival analysis demonstrated a positive association between high MII and an increased cumulative incidence of mortality and MACCE (Figure 2). This relationship was found to be statistically significant based on the log-rank test (*P* < 0.001). The association of a high level of inflammatory indices such as NLR, PLR, SII, CRP, and MII with both mortality and MACCE was found to be statistically significant in both univariate and multivariate Cox proportional hazard analyses (*P* < 0.001, each). Among those, high MII demonstrated a relatively higher hazard ratio than other inflammatory markers such as NLR, PLR, SII and CRP. The results remained consistent across both PS-matched and IPW-adjusted analyses (Table 4). Covariate balance was assessed using standardized mean differences (SMDs), with all matched variables achieving SMDs within ±10%, indicating successful balance (Table 5).

**Table 3:** Comparison of adverse events during follow-up between high MII group and Low MII group.

Adverse events	High MII group (*n* = 3,708)	Low MII group (*n* = 4,706)	*p*-value
Overall death (%)	1,110 (29.9)	608 (12.9)	<0.001
MACCE (%)	1,350 (36.4)	892 (19.0)	<0.001
MI (%)	241 (6.5)	200 (4.2)	<0.001
Stroke (%)	177 (4.8)	188 (4.0)	0.082

Data are presented as *n* (%) for categorical variables. MACCE indicates major adverse cardiac and cerebrovascular events; MI, myocardial infarction.

**Table 4:** Impact of inflammatory markers on risk of mortality and MACCE in acute myocardial patients.

Group	No death (*n* = 6,696)	Death (*n* = 1,718)	Unadjusted		Multivariable-adjusted		Propensity-score matched		IPW-adjusted	
			HR (95% CI)	*p*-value	HR (95% CI)	*p*-value	HR (95% CI)	*p*-value	HR (95% CI)	*p*-value
High NLR	2,070 (30.9)	786 (45.8)	1.82 (1.65–2.00)	<0.001	1.40 (1.27–1.54)	<0.001	1.27 (1.14–1.41)	<0.001	1.27 (1.14–1.41)	<0.001
High PLR	2,213 (33.0)	831 (48.4)	1.75 (1.59–1.92)	<0.001	1.29 (1.17–1.42)	<0.001	1.18 (1.06–1.31)	<0.001	1.17 (1.06–1.30)	<0.001
High CRP	2,679 (40.0)	1,091 (63.5)	2.20 (2.00–2.43)	<0.001	1.61 (1.46–1.78)	<0.001	1.69 (1.52–1.87)	<0.001	1.68 (1.51–1.86)	<0.001
High SII	2,266 (33.8)	803 (46.7)	1.67 (1.52–1.84)	<0.001	1.37 (1.24–1.51)	<0.001	1.28 (1.15–1.42)	<0.001	1.28 (1.15–1.42)	<0.001
High MII	2,598 (38.8)	1,110 (64.6)	2.43 (2.20–2.68)	<0.001	1.71 (1.55–1.89)	<0.001	1.61 (1.45–1.79)	<0.001	1.60 (1.44–1.78)	<0.001
Group	No MACCE (*n* = 6,172)	MACCE (*n* = 2,242)	Unadjusted		Multivariable-adjusted		Propensity-score matched		IPW-adjusted	
			HR (95% CI)	*p*-value	HR (95% CI)	*p*-value	HR (95% CI)	*p*-value	HR (95% CI)	*p*-value
High NLR	1,900 (30.8)	956 (42.6)	1.60 (1.48–1.74)	<0.001	1.31 (1.20–1.42)	<0.001	1.19 (1.08–1.31)	<0.001	1.19 (1.08–1.31)	<0.001
High PLR	2,038 (33.0)	1,006 (44.9)	1.53 (1.40–1.70)	<0.001	1.18 (1.09–1.29)	<0.001	1.13 (1.03–1.24)	<0.001	1.13 (1.02–1.24)	<0.001
High CRP	2,437 (39.5)	1,333 (59.5)	1.87 (1.72–2.03)	<0.001	1.44 (1.32–1.57)	<0.001	1.52 (1.39–1.66)	<0.001	1.52 (1.38–1.66)	<0.001
High SII	2,080 (33.7)	989 (44.1)	1.51 (1.39–1.64)	<0.001	1.28 (1.18–1.40)	<0.001	1.21 (1.10–1.33)	<0.001	1.20 (1.10–1.32)	<0.001
High MII	2,358 (38.2)	1,350 (60.2)	2.03 (1.87–2.21)	<0.001	1.53 (1.4–1.67)	<0.001	1.59 (1.45–1.74)	<0.001	1.59(1.45–1.74)	<0.001

Data are presented as *n* (%) for categorical variables. The variables of multivariate analysis: old age, gender, high blood pressure, diabetes mellitus, smoking, previous coronary artery bypass graft, previous stroke, anemia, chronic kidney disease, chronic lung disease, medications at discharge. The variables considered in propensity matching: old age, sex, hypertension, diabetes, smoking, severe anemia, chronic kidney disease, chronic lung disease, history of coronary artery bypass grafting and stroke, and medications at discharge. Old age defined as age ≥65. IPT inverse probability weighting; NLR, neutrophil to lymphocyte ratio; PLR, platelet to lymphocyte ratio; CRP, C-reactive protein; SII, systemic immune-inflammatory index; MII, multi-inflammatory index;.

**Table 5:** Baseline characteristics of study population after propensity score matching.

Variables	High MII (*n* = 3,433)	Low MII (*n* = 3,433)	*p*-value	Absolute SMD
Age (yrs)	64.8 ± 12.7	64.3 ± 12.2	0.108	0.039
Gender, female (%)	2,397 (69.8)	2,464 (71.8)	0.075	0.043
HBP (%)	1,869 (54.4)	1,838 (53.5)	0.453	0.018
DM (%)	1,185 (34.5)	1,143 (33.3)	0.284	0.026
Dyslipidemia (%)	559 (16.3)	559 (16.3)	1.000	<0.001
Smoking (%)	1,830 (53.3)	1,893 (55.1)	0.127	0.037
Previous MI (%)	135 (3.9)	148 (4.3)	0.430	0.019
Previous PCI (%)	241 (7.0)	248 (7.2)	0.743	0.008
Previous CABG (%)	17 (0.5)	15 (0.4)	0.723	0.009
Previous stroke (%)	282 (8.2)	236 (6.9)	0.036	0.051
Cancer (%)	112 (3.3)	126 (3.7)	0.356	0.022
Chronic lung disease (%)	93 (2.7)	85 (2.5)	0.543	0.015
Chronic liver disease (%)	24 (0.7)	36 (1.0)	0.120	0.038
Anemia (%)	363 (10.6)	282 (8.2)	0.001	0.081
CKD (%)	69 (2.0)	50 (1.5)	0.079	0.042
Laboratory findings				
White blood cell (10^9^/L)	12.7 ± 7.2	10.3 ± 5.6	<0.001	0.370
Neutrophil (10^9^/L)	8.7 ± 13.9	6.1 ± 3.2	<0.001	0.259
Lymphocyte (10^9^/L)	2.6 ± 2.0	3.0 ± 2.0	<0.001	0.211
Platelet (10^9^/L)	234.0 ± 71.4	229.3 ± 62.7	0.004	0.070
C-reactive protein (mg/L)	77.5 ± 190.6	2.4 ± 3.2	<0.001	0.558
NLR	5.1 ± 6.1	2.8 ± 2.4	<0.001	0.497
PLR	138.0 ± 112.4	103.6 ± 75.0	<0.001	0.360
SII	1,200.8 ± 1,579.3	640.9 ± 626.	<0.001	0.466
MII	362.6 ± 1,700.9	4.9 ± 4.4	<0.001	0.297
Medication at discharge				
Aspirin (%)	3,374 (98.3)	3,381 (98.5)	0.503	0.016
Clopidogrel (%)	3,106 (90.5)	3,110 (90.6)	0.869	0.004
Potent P2Y12 inhibitor (%)	307 (8.9)	307 (8.9)	1.000	<0.001
Oral anticoagulant (%)	97 (2.8)	93 (2.7)	0.769	0.007
Beta-blockers (%)	2,982 (86.9)	3,022 (88.0)	0.145	0.035
Statins (%)	3,317 (96.6)	3,335 (97.1)	0.211	0.030

Data are presented as mean ± standard deviation for continuous variables and *n* (%) for categorical variables. Anemia defined as hemoglobin <13.5 mg/dl for males and <12 mg/dl for females. CKD defined as estimated glomerular filtration rate <60 ml/min/1.73 m^2^. The variables considered in propensity matching: age, sex, hypertension, diabetes, smoking, severe anemia, chronic kidney disease, chronic lung disease, history of coronary artery bypass grafting and stroke, and medications at discharge. SMD indicates standard mean difference; HBP, high blood pressure; DM, diabetes mellitus; MI, myocardial infarction; PCI, percutaneous coronary intervention; CABG, coronary artery bypass graft; CKD, chronic kidney disease; NLR, neutrophil to lymphocyte ratio; PLR, platelet to lymphocyte ratio; SII, systemic immune-inflammatory index; MII, multi-inflammatory index.

In the ROC curve analysis, the AUC of MII for predicting mortality was 0.662 while the AUC of CRP (a conventional, well-known inflammatory marker) was 0.646, showing a statistically significant difference (*P* < 0.001) (Table 2). The inclusion of a high MII level alongside traditional risk factors (Model B) demonstrated a significantly improved reclassification ability for predicting all-cause mortality compared to the model consisting of traditional risk factors alone (Model A) (C-index 0.791 vs. 0.780; 95% CI 0.780–0.803; *P* < 0.001, NRI 0.018; 95% CI 0.014–0.021; *P* < 0.001) (Table 6). The same trend was shown in prediction models for MACCE (Model B' vs. Model A', C-index 0.743 vs. 0.733, *P* < 0.001, NRI 0.014; 95% CI 0.011–0.017; *P* < 0.001).

**Table 6:** Effects of variables on the prediction accuracy and risk reclassification of each model (traditional risk factors only vs. traditional risk factors + high MII).

Models		C-index	95% CI	*p*-value	NRI	95% CI	*p*-value
Models for predicting mortality							
Model A	Old age, gender, HBP, DM, anemia, CKD, smoker, history of CABG, stroke, chronic lung disease	0.780	0.768–0.792				
Model B (vs. A)	Model A + high MII	0.791	0.78–0.803	<0.001	0.018	0.014–0.021	<0.001
Models for predicting MACCE							
Model A’	Old age, gender, HBP, DM, anemia, CKD, smoker, history of CABG, stroke, chronic lung disease	0.733	0.721–0.745				
Model B’ (vs. A’)	Model A’ + high MII	0.743	0.731–0.755	<0.001	0.014	0.011–0.017	<0.001

Old age defined as age ≥65. CKD defined as estimated glomerular filtration rate <60 ml/min/1.73 m^2^. Anemia defined as hemoglobin <13.5 mg/dl for males and <12 mg/dl for females. NRI indicates net reclassification index; CI, confidence interval; HBP, high blood pressure; DM, diabetes mellitus; CKD, chronic kidney disease; CABG, coronary artery bypass graft; MII, multi-inflammatory index.

**Figure 3:**
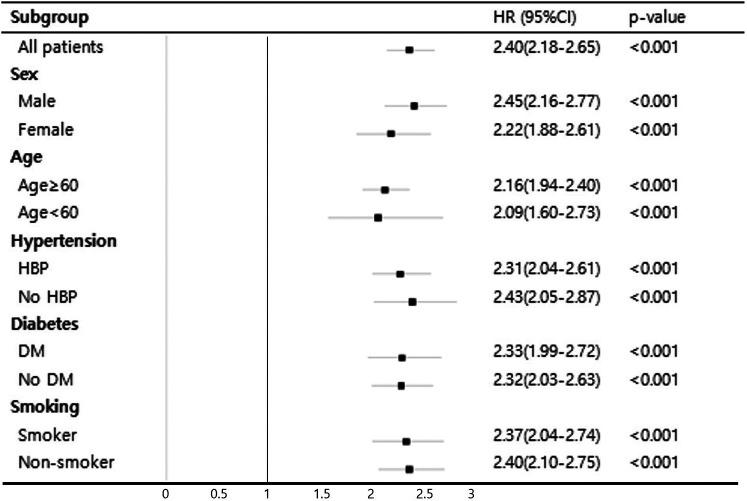
Subgroup Cox proportional hazard analysis of high MII for mortality.

Subgroup analyses based on traditional cardiovascular risk factors that showed significant differences in baseline characteristics including sex, age, hypertension, diabetes, and smoking history are presented in Figure 3. Notably, a significant association between high MII and mortality was observed across all subgroups (*p* < 0.001).

## Discussion

The results of our study indicate that in the case of patients successfully treated with PCI after a myocardial infarction, those with a high MII score have a significantly greater risk of mortality and MACCE than those with a low MII score.

Inflammation plays a significant role in the development of CAD, particularly in acute coronary syndrome (ACS). Many researchers have suggested that certain inflammatory markers can predict the prognosis of cardiovascular diseases and have been working to better understand the relevant indicators. So far, what has been found among leukocyte subsets to be the most valuable indicator of cardiovascular mortality are neutrophils, which are known to mediate the early inflammatory response following a myocardial infarction (12). This is attributed, in part, to the serum ingredients produced by neutrophils, such as gelatinases, collagenases, and elastase (13). As a consequence, the endothelial layer undergoes activation and dysregulation, leading to the extravasation of low density lipoprotein cholesterol and facilitating the formation of foam cells. Additionally, reduced lymphocyte levels have been observed to be correlated to unfavorable cardiovascular outcomes in ACS (14). For example, lymphocytopenia is independently linked to the occurrence of mechanical complications following a myocardial infarction (15). Furthermore, patients with heart failure who exhibit lower lymphocyte counts experience higher mortality rates (16). Additionally, a combination of serum albumin and CRP has been studied in patients with coronary artery disease who have undergone PCI, where a findings showed that higher CAR was found to be associated with an increased risk of mortality (17).

However, while there are several inflammatory markers currently known to be associated with patient prognosis in CAD, relying on a single inflammatory marker is insufficient to accurately predict the extent or severity of inflammation. Yang et al. (18) reported that SII (an inflammatory indicator composed of neutrophil, platelet, and lymphocyte counts) was a superior predictor of MACCE compared to traditional risk factors in CAD patients. Another group of researchers reported that SII was independently associated with poor prognosis in acute MI patients who underwent PCI (19). In our study, we focused on a novel systemic inflammation index called MII, which was first introduced by Gardini et al. (10), consisting of a combination of neutrophils, lymphocytes, and CRP. They confirmed the utility of this novel index in assessing the prognosis of patients with colorectal cancer. Other researchers similarly demonstrated the efficacy of this marker as a predictive tool for mortality in patients with COVID-19 (11). Additionally, a recent study has proposed that this index plays a significant role in distinguishing between pulmonary embolism cases classified as massive or non-massive (20). Similarly, MII has also been shown to be helpful in early detection of poor prognosis in acute ischemic stroke (21). However, the potential prognostic value of MII in the field of cardiology has not been previously evaluated. In doing so for our study, we chose MII as the independent variable and observed a significant association between high MII levels and poor prognosis.

In the CANTOS trial, the indicator used for inflammation was CRP, with patients receiving Canakinumab if they had a CRP level of 2 mg/L or more. Canakinumab, an antibody that functions by inhibiting interleukin-1b, has been observed to decrease CRP in a dose-dependent manner and has shown efficacy in reducing cardiovascular events (22). The CRP level is a simple and easily accessible measure, but we believe that MII is similarly easy enough to obtain while also reflecting the inflammatory state better and offering improved predictive capabilities for mortality. From our results, in terms of discriminative ability for mortality, high MII was superior over a simple CRP level, as evidenced by a significantly larger AUC in the ROC curve analysis. Additionally, among the examined inflammatory markers in this study such as NLR, PLR, and SII, MII provided a relatively higher hazard ratio, indicating stronger association with the outcome. Moreover, the addition of high MII levels to the traditional risk factors (Model B, C-index 0.791) significantly enhanced the ability to reclassify and predict all-cause mortality compared to the model with traditional risk factors alone (Model A, C-index 0.780) (*p* < 0.001) (Table 6). This finding suggests that the incorporation of high MII levels adds valuable predictive information beyond what is provided by traditional risk factors alone.

For a more accurate treatment strategy and medical resource allocation, individualized and timely risk assessment is necessary for each patient with acute MI. Complete blood count and serum CRP tests are quick, easy, and cost-effective methods for evaluating the risk of morbidity and mortality for these patients. The combination of these inflammation indices can rapidly give an indication of the risk associated with post-MI patients, complement the traditional prognosis predictors, and individualize the treatment. This can also be useful in situations where further imaging or laboratory tests are not available, as long as a complete blood count and a biochemistry test can be performed. This method of instant risk assessment can be especially beneficial to areas with limited medical resources.

In this study, MII demonstrated strong discriminative and reclassification power for predicting mortality and MACCE in acute MI patients treated with PCI. When compared to other inflammatory markers such as CRP, NLR, PLR, and SII, MII displayed a significantly greater AUC in the ROC curve analysis (Table 2) and a relatively higher hazard ratio in the Cox proportional analysis (Table 4). These findings suggest the significant association between high MII and all-cause mortality or MACCE. Table 6 illustrates the incremental value of high MII levels over traditional risk factors by using standard metrics of model performance. Adding high MII to the traditional risk factor combination models significantly improved the predictive accuracy for discrimination. Furthermore, the augmented model (Model B or B') demonstrated significantly enhanced performance in predicting the probability of re-classification. Subgroup analysis further supported the association between MII and long-term clinical outcomes, revealing statistically significant hazard ratios across all subgroups and confirming its effectiveness in predicting mortality in acute MI patients treated with PCI. The influential CANTOS trial played a crucial role in advancing the inflammatory hypothesis of coronary artery disease. Based on our study's results, we believe that MII has the possibility to serve as a superior inflammatory marker compared to conventional markers in future studies aiming to improve the prognosis of acute MI patients using anti-inflammatory treatment mechanisms.

This study has several limitations. It is important to note that this study was focused on the Korean population; consequently, there was a lack of external validation of MII in other populations. The generalizability of these findings should be approached with caution. Also, due to the absence of continuous monitoring of blood tests in this study, the assessment of MII was limited to a single time point, and fluctuations in MII were not taken into account. The discriminatory power of the inflammatory indices, as reflected by the C-index values shown in Table 2, was relatively modest. This may suggest potential limitations in generalizability due to unmeasured confounding, measurement error, or heterogeneity among subpopulations. However, combining it with traditional risk factors have shown significantly improved recalssification ability for predicting mortality as shown in Table 6. Lastly, detailed angiographic features such as lesion length, bifurcation involvement, and presence of restenosis were not consistently documented, limiting our ability to assess procedural complexity. Similarly, data on the use of drug-coated balloons (DCBs) were not uniformly recorded. These gaps reflect the limitations of retrospective data collection and may have led to residual confounding. However, to control for potential confounding factors between the groups, we utilized both PS matching and IPW, and these methods consistently demonstrated statistically significant findings regarding the prognostic value of MII.

## Conclusion

While aggressive lipid-lowering therapy with statins has become the standard recommendation for patients with acute coronary syndromes, anti-inflammatory therapy is seen as the next challenge to be addressed. In the present study, we investigated the utility of a novel biomarker score, MII, as an alternative to the traditional inflammatory markers, which have limited specificity for myocardial infarction. In our study with AMI patients who received successful reperfusion treatment, 29.9% of patients with high MII died over five years, and a significantly higher all-cause mortality rate was observed compared to the low MII population. Additionally, high MII showed an association with long-term clinical outcomes and showed significantly higher performance compared to conventional inflammatory markers including CRP. Moreover, high MII has resulted in significant improvements in discrimination and reclassification of risk prediction by adding to previously well-known traditional disease risk factors.

## Data Availability

The datasets are not publicly available due to patient privacy concerns and institutional data sharing policies, but they can be obtained from the corresponding author upon reasonable request.
